# Positron Emission Tomography Imaging of *Acinetobacter baumannii* Infection: Comparison of Gallium-68 Labeled Siderophores

**DOI:** 10.1021/acsinfecdis.4c00946

**Published:** 2025-03-18

**Authors:** Katerina Dvorakova Bendova, Kristyna Krasulova, Barbora Neuzilova, Miroslav Popper, Patrik Mlynarcik, Katarina Hajduova, Zbynek Novy, Marian Hajduch, Milos Petrik

**Affiliations:** † Institute of Molecular and Translational Medicine, Faculty of Medicine and Dentistry, Palacký University, 779 00 Olomouc, Czech Republic; ‡ Department of Microbiology, Faculty of Medicine and Dentistry, Palacký University and University Hospital, 775 15 Olomouc, Czech Republic; § Laboratory of Experimental Medicine, University Hospital, 779 00 Olomouc, Czech Republic; ∥ Czech Advanced Technology and Research Institute, Palacký University, 779 00 Olomouc, Czech Republic

**Keywords:** siderophores, radiolabeling, gallium-68, PET, Acinetobacter baumannii

## Abstract

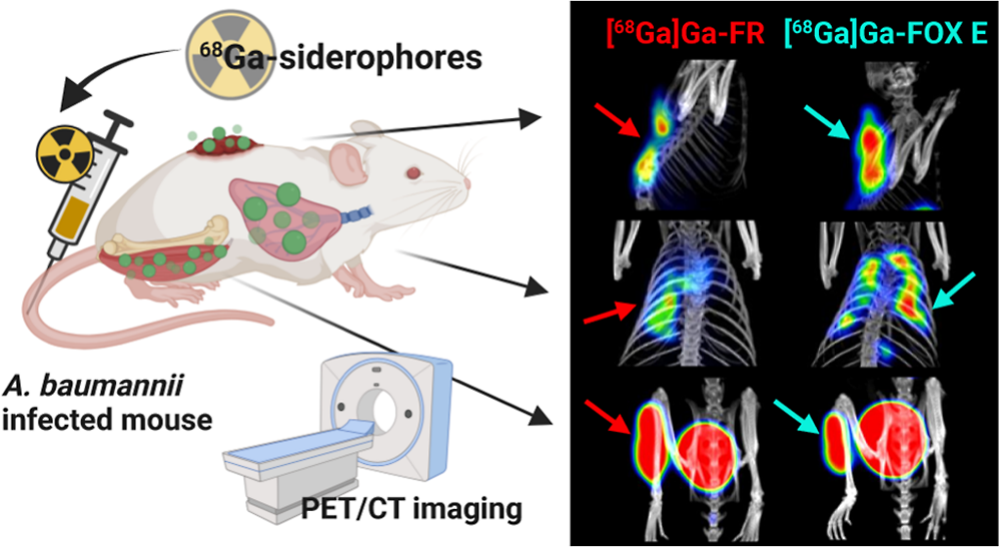

*Acinetobacter baumannii* (AB) is an opportunistic pathogen with growing clinical relevance due to its increasing level of antimicrobial resistance in the last few decades. In the event of an AB hospital outbreak, fast detection and localization of the pathogen is crucial, to prevent its further spread. However, contemporary diagnostic tools do not always meet the requirements for rapid and accurate diagnosis. For this reason, we report here the possibility of using gallium-68 labeled siderophores, bacterial iron chelators, for positron emission tomography imaging of AB infections. In our study, we radiolabeled several siderophores and tested their in vitro uptake in AB cultures. Based on the results and the in vitro properties of studied siderophores, we selected two of them for further in vivo testing in infectious models. Both selected siderophores, ferrioxamine E and ferrirubin, showed promising in vitro characteristics. In vivo, we observed rapid pharmacokinetics and no excessive accumulation in organs other than the excretory organs in normal mice. We demonstrated that the radiolabeled siderophores accumulate in AB-infected tissue in three animal models: a murine model of myositis, a murine model of dorsal wound infection and a rat model of pneumonia. These results suggest that both siderophores radiolabeled with Ga-68 could be used for PET imaging of AB infection.

*Acinetobacter baumannii* (AB) is a Gram-negative, obligate aerobic bacterium that is ubiquitous in many environments and is a normal coloniser of living organisms.^1^ Since the mid 1990s, when the clinical relevance of this pathogen was severely underrated, AB has emerged as an important agent of hospital-acquired infections (HAI).^2,3^ Nowadays, AB’s capacity to survive desiccation and disinfectants, its ability of forming biofilms on medical equipment and increasing resistance to known antibiotics have brought this microbe to the forefront of medical and research interest.^4^ The gravity of the situation is underlined by the fact, that in 2018, carbapenem-resistant AB was listed by World Health Organization as one of the three bacterial pathogens of critical priority for research and development of new drugs.^5^ In addition, colistin resistance, especially in AB, varies globally. It surged during the 2019–2020 pandemic, particularly in Western Europe. The discovery of mobile colistin resistance calls for close monitoring of this pathogen.^6^ Even though AB is capable of causing community-acquired infections, mainly in people with pre-existing comorbidities living in humid regions, the majority of infections caused by AB are HAIs. Globally, AB is isolated from more than 20% of all nosocomial infections and it is the most common infectious agent in patients admitted to intensive care units.^7^ It causes a variety of infections, often associated with indwelling devices or surgical procedures. It invades the bloodstream, surgical wounds and the urinary tract. However, the most prominent AB infection is pneumonia, which is often associated with mechanical ventilation and is linked to higher mortality rates.^8−10^

During a hospital outbreak of AB, it is important to quickly identify the infected patients to prevent further spread through the medical environment.^11^ It is then essential to accurately diagnose and treat these patients with targeted antibiotics, as empiric antibiotic use is associated with increased mortality.^12^ However, gold standard diagnostic methods do not always meet these requirements. For example, culture-based assays are usually time-consuming and molecular techniques may fail to distinguish between colonisation or infection.^13,14^ To improve the accuracy of these methods, invasive sampling is often used. While this may indeed reduce the initial antibiotic burden on the patient, it is linked to increased risks during these procedures, which are particularly dangerous for critically ill patients.^15^

Given the shortcomings of available methods, there is an urgent need for a novel tool that can overcome these difficulties. In this regard, siderophores may prove useful in the diagnosis of infections. These low-molecular-weight chelators are produced by diverse range of organisms, including fungi, plants and bacteria. As the main function of siderophores in bacteria is the scavenging of iron, which is essential for their survival, basic metabolism and various other processes (e.g., biofilm formation, toxin synthesis), their production is strongly influenced by the availability of the iron in the environment.^16−18^ Thanks to the high affinity of siderophores, some are able to remove the iron from the host molecule and capture it for themselves.^17^ The scarcity of iron available during an infection forces bacteria to compete not only with the host organism but also with each other. To overcome rival pathogens, some bacteria are able to utilize xenosiderophores, meaning they can benefit from siderophores produced by other strains without producing them themselves.^19^ Even though AB produces several of its own siderophores, previous research has shown that AB is also able to utilize xenosiderophores.^20,21^

Since iron bound in siderophores has similar physical-chemical properties to gallium-68, the binding of these elements to siderophores is interchangeable. As gallium-68 is a positron emitter with a short half-life and is easily accessible from an on-site generator, there is a great opportunity to radiolabel siderophores with gallium-68 and use this conjugation for the detection of AB by positron emission tomography (PET).^22^ Previous studies demonstrated that radiolabeled siderophores might be used for detection of microbial pathogens.^23−25^ In this work we aim to demonstrate that the same principle can be used to image AB infection, thus providing a novel diagnostic tool for AB infection. This work focuses on two xenosiderophores, ferrioxamine E (FOX E) and ferrirubin (FR) (Figure 1A,B respectively), radiolabeled with Ga-68, which were selected based on their favorable in vitro properties explored in previous studies and on the initial results of this work.^26,27^

**Figure 1:**
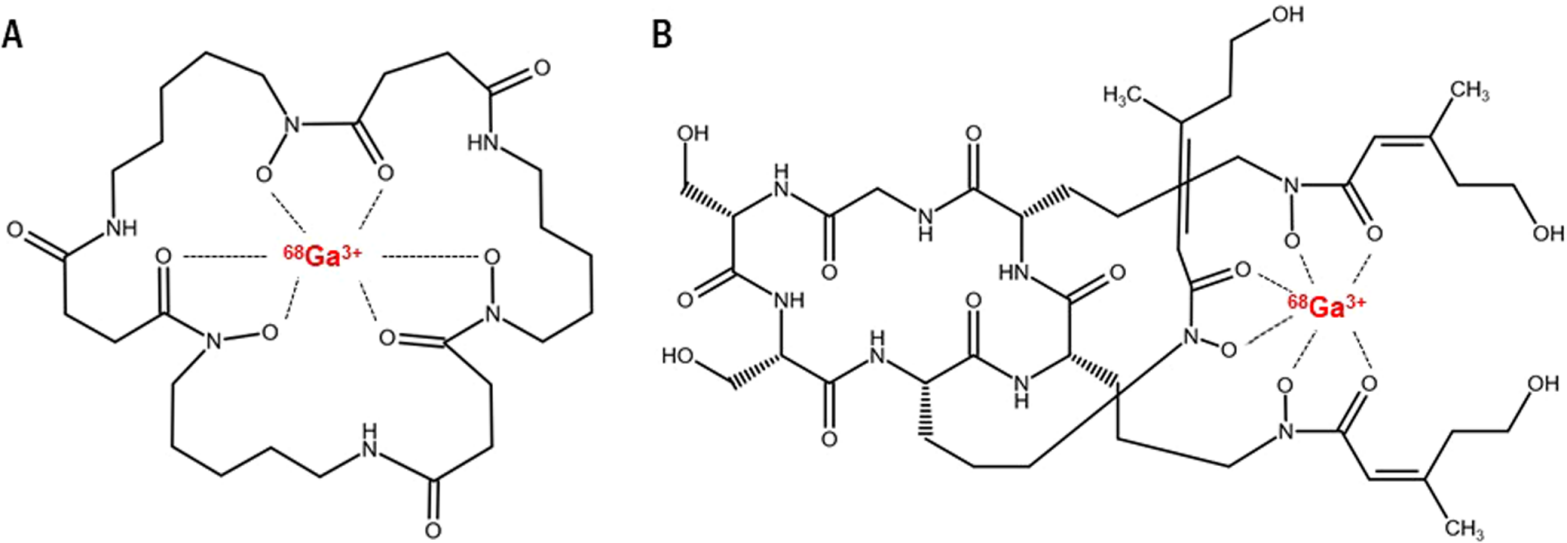
Chemical structures of tested siderophores. (A) [^68^Ga]Ga-ferrioxamine E. (B) [^68^Ga]Ga-ferrirubin.

## Results

### Radiolabeling and Quality Control of [^68^Ga]Ga-FOX E and [^68^Ga]Ga-FR

Most siderophores used in the study were radiolabeled with gallium-68 with high radiochemical purity (>95%) confirmed by either RP-radioHPLC or radio-iTLC. The only exception was the [^68^Ga]Ga-FR, which reached slightly lower values (>91%). The radiochemical purity of [^68^Ga]Ga-FOX E and [^68^Ga]Ga-FR was confirmed by both methods (Figure S1A,B).

### In Vitro Uptake Assays of Radiolabeled Siderophores in AB Cultures

**Figure 2:**
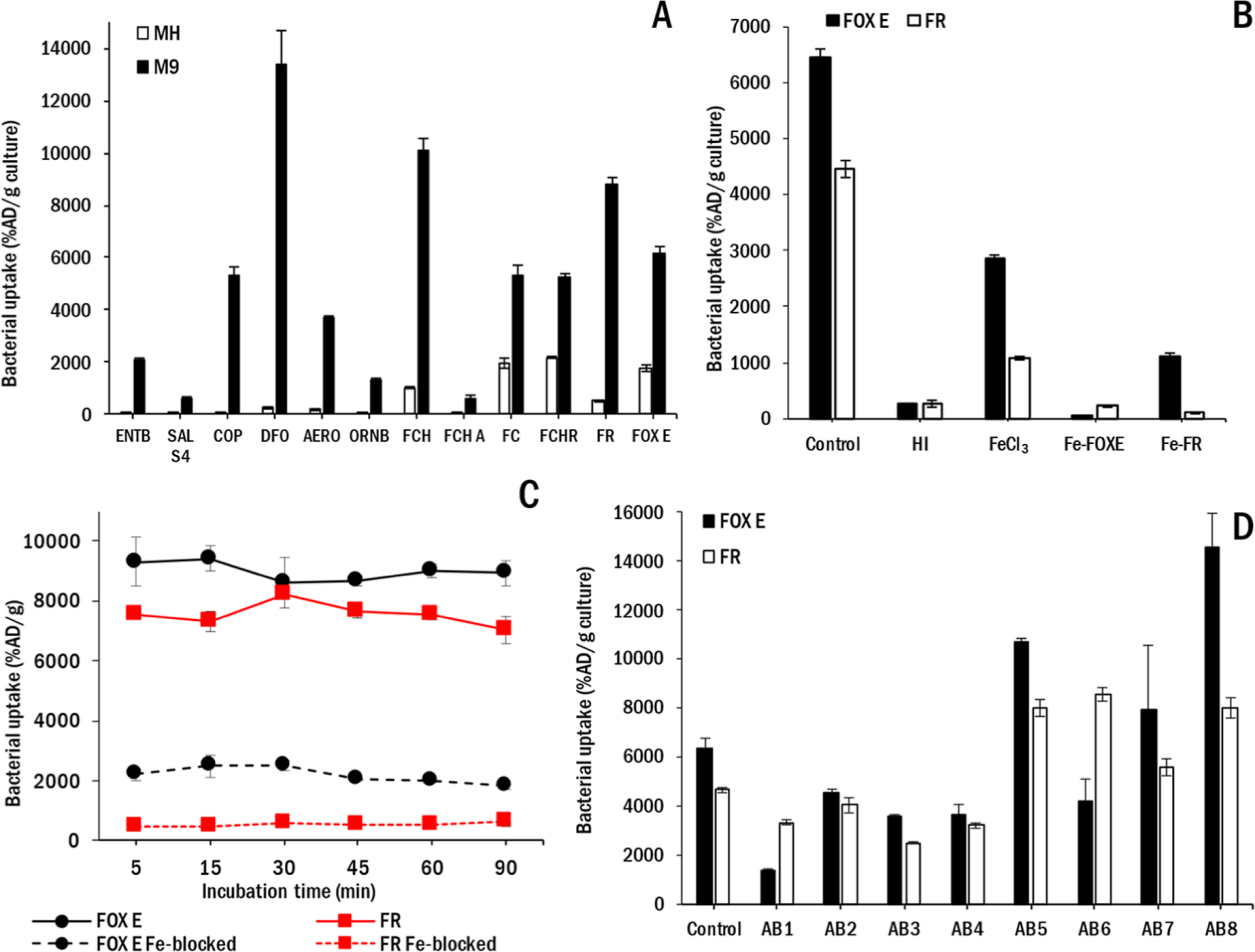
(A) Comparison of in vitro uptake of various gallium-68 labeled siderophores after 45 min of incubation in *Acinetobacter baumannii* NCTC13301 grown in MH (white) or M9 (black) medium (ENTB = [^68^Ga]Ga-enterobactin; SAL S4 = [^68^Ga]Ga-salmochelin S4; COP = [^68^Ga]Ga-coprogen; DFO = [^68^Ga]Ga-desferrioxamine B; AERO = [^68^Ga]Ga-aerobactin; ORNB = [^68^Ga]Ga-ornibactin; FCH = [^68^Ga]Ga-ferrichrome; FCH A = [^68^Ga]Ga-ferrichrome A; FC = [^68^Ga]Ga-ferricrocin; FCHR = [^68^Ga]Ga-ferrichrysin; FR = [^68^Ga]Ga-ferrirubin; FOX E = [^68^Ga]Ga-ferrioxamine E). (B) Uptake comparison of radiolabeled siderophores in heat-inactivated AB culture and AB cultures preincubated with excess of FeCl_3_, Fe-FOXE or Fe-FR grown in M9 medium (control = AB culture without preincubation; HI = heat-inactivated AB culture; FeCl_3_ = AB culture preincubated with excess of FeCl_3_; Fe-FOXE = AB culture preincubated with Fe-FOXE; Fe-FR = AB culture preincubated with Fe-FR). (C) Comparison of in vitro uptake of [^68^Ga]Ga-FOX E and [^68^Ga]Ga-FR in AB culture in time. Interrupted lines represent the uptake of siderophores in AB culture cultivated in M9 medium with excess of iron. (D) Comparison of in vitro uptake of [^68^Ga]Ga-FOX E and [^68^Ga]Ga-FR in various clinically acquired samples of *Acinetobacter baumannii* grown in M9 medium (control = *Acinetobacter baumannii* NCTC13301; AB1 = *A. baumannii* 9022/c; AB2 = *A. baumannii* 7948/c; AB3 = *A. baumannii* 6535/a; AB4 = *A. baumannii* 11069/A; AB5 = *A. baumannii* 8905/c; AB6 = *A. baumannii* 13515/a; AB7 = *A. baumannii* 17807/a; AB8 = *A. baumannii* 20192/c).

Overall, the majority of the radiolabeled siderophores tested showed uptake depending on growth conditions of the AB culture. Most of the siderophores showed particularly high uptake in the culture that was grown in M9 medium ([^68^Ga]Ga-desferrioxamine B, [^68^Ga]Ga-ferrichrome, [^68^Ga]Ga-ferrirubin, [^68^Ga]Ga-ferrioxamine E, [^68^Ga]Ga-ferricrocin, [^68^Ga]Ga-ferrichrysin, [^68^Ga]Ga-coprogen, [^68^Ga]Ga-aerobactin and [^68^Ga]Ga-enterobactin). On the other hand, only a few siderophores showed some uptake (>500%AD/g culture) in the AB culture grown in MH medium, specifically only ([^68^Ga]Ga-ferrichrysin, [^68^Ga]Ga-ferricrocin, [^68^Ga]Ga-ferrioxamine E, [^68^Ga]Ga-ferrichrome and [^68^Ga]Ga-ferrirubin) (Figure 2A). Based on these results and factors that are further explained in the discussion, we selected [^68^Ga]Ga-FR and [^68^Ga]Ga-FOX E for further testing.

The uptake of both ^68^Ga-siderophores could be blocked by heat inactivation of the bacterial culture. However, the uptakes of [^68^Ga]Ga-FR and [^68^Ga]Ga-FOX E were only partially blocked by preincubation of the culture with FeCl_3_ (the uptake decreased by 76% and 56% for both ^68^Ga-siderophores respectively). Both ^68^Ga-siderophores were successfully blocked by preincubation of the culture with excess of Fe-FOX E (decrease by 99% for [^68^Ga]Ga-FOX E and by 95% for [^68^Ga]Ga-FR), but the uptake of [^68^Ga]Ga-FOX E appeared to be less affected by preincubation of the culture with Fe-FR than [^68^Ga]Ga-FR (decrease by 83% and 98% respectively) (Figure 2B). Both ^68^Ga-siderophores reached high uptake levels in the AB culture as early as 5 min after incubation and neither ^68^Ga-siderophore showed significant increase or decrease in uptake over time in both normal cultures and cultures preincubated with relevant iron–siderophore complex (Figure 2C). The in vitro assays revealed an overall high level of uptake by the AB culture in all of the clinical samples tested. The uptake of both ^68^Ga-siderophores varied in each culture and neither [^68^Ga]Ga-FOX E nor [^68^Ga]Ga-FR had a generally higher uptake than the other ^68^Ga-siderophore (Figure 2D).

### Animal Imaging Studies

**Figure 3:**
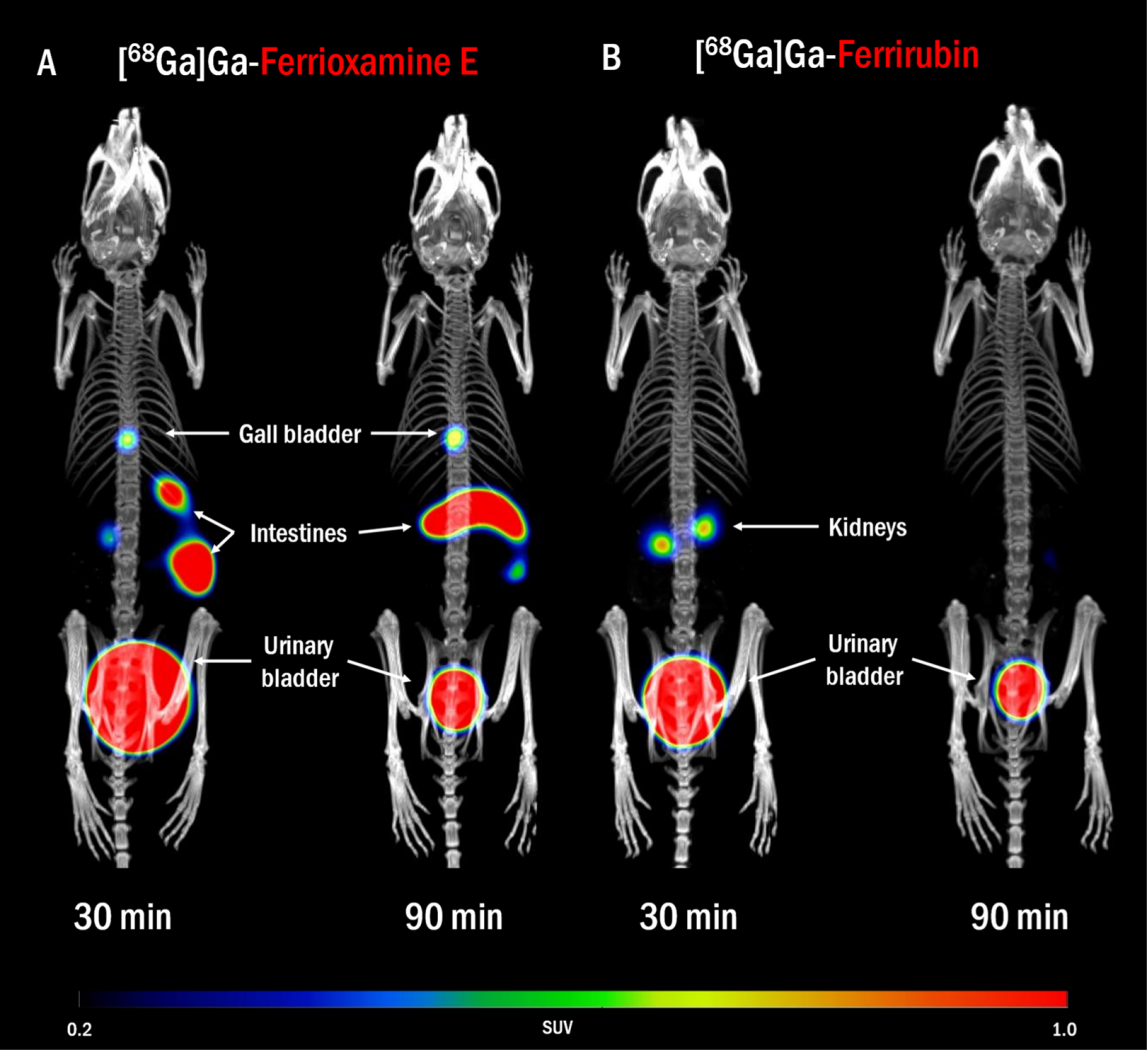
Maximum intensity projection (MIP) PET/CT images of in vivo biodistribution of (A) [^68^Ga]Ga-FOX E and (B) [^68^Ga]Ga-FR in healthy mice 30 and 90 min after radiolabeled siderophore administration.

A biodistribution study in noninfected Balb/c mice showed rapid biodistribution with fast clearance from the blood for both radiolabeled siderophores with predominantly renal excretion. [^68^Ga]Ga-FR showed no accumulation in major organs, but in the case of [^68^Ga]Ga-FOX E we observed some activity in the gallbladder and intestine (Figure 3). These results are in accordance with previously published studies.^26,27^

**Figure 4:**
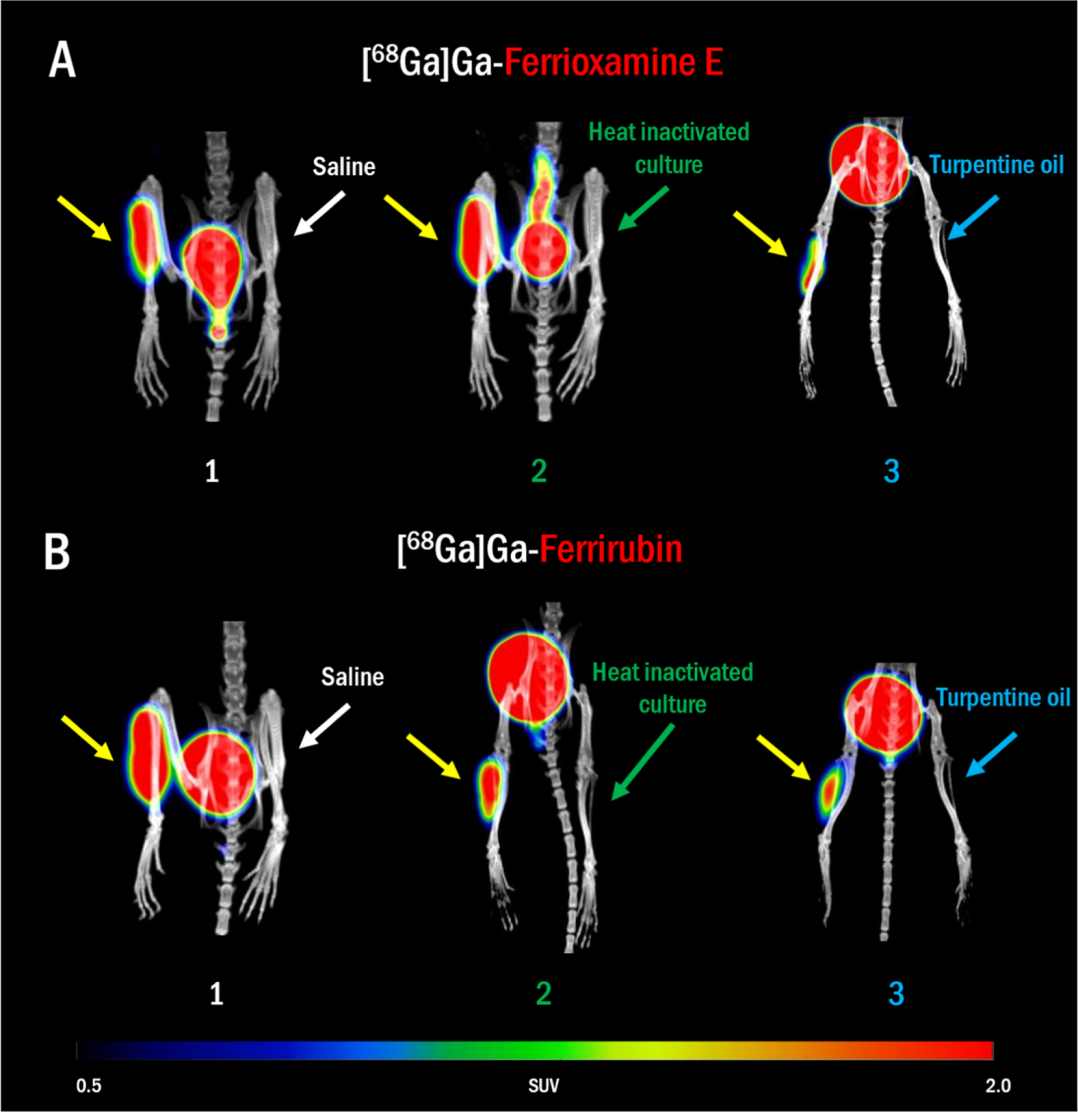
PET/CT in vivo imaging of (A) [^68^Ga]Ga-FOX E and (B) [^68^Ga]Ga-FR in murine model of myositis in the left hind leg induced by AB NCTC 13301 (yellow arrows) and control in the right hind leg: (1) saline (white arrows), (2) heat inactivated AB culture (green arrows) and (3) turpentine oil (blue arrows). The imaging was performed 5 h after infection and 45 min after radiolabeled siderophore administration. MIP images.

In a murine model of AB-induced acute myositis induced 5 h before imaging we observed high accumulation of signal in the infected leg for both ^68^Ga-siderophores tested and no signal accumulation in the legs injected with control substances (Figure 4). Quantitative analysis revealed a significant difference in mean SUVs between infected and noninfected legs for both ^68^Ga-siderophores (*P* < 0.001) (Figure S4A). In a murine model of myositis comparing different infectious doses (Figure S2), both ^68^Ga-siderophores showed reliable uptake at the dose of 8 × 10^6^ CFU. [^68^Ga]Ga-FOX E also showed a moderate level of accumulation in the tissue infected with 8 × 10^5^ CFU, while the signal for [^68^Ga]Ga-FR was much lower. We observed only a low level of [^68^Ga]Ga-FOX E accumulation at the 8 × 10^4^ dose, suggesting the limit of detection around 9 × 10^2^ pathogen/mm^3^. In the dynamic study of AB-induced murine myositis, we observed significant uptake in the infected leg as early as in the first time frame with both ^68^Ga-siderophores and the uptake was increasing in time (Figure S3A1,A2). On the time–activity curves created from the dynamic studies, we also observed an increase in signal uptake in the infected leg and a decrease of the activity in the control, noninfected leg over time (Figure S3B1,B2).

**Figure 5:**
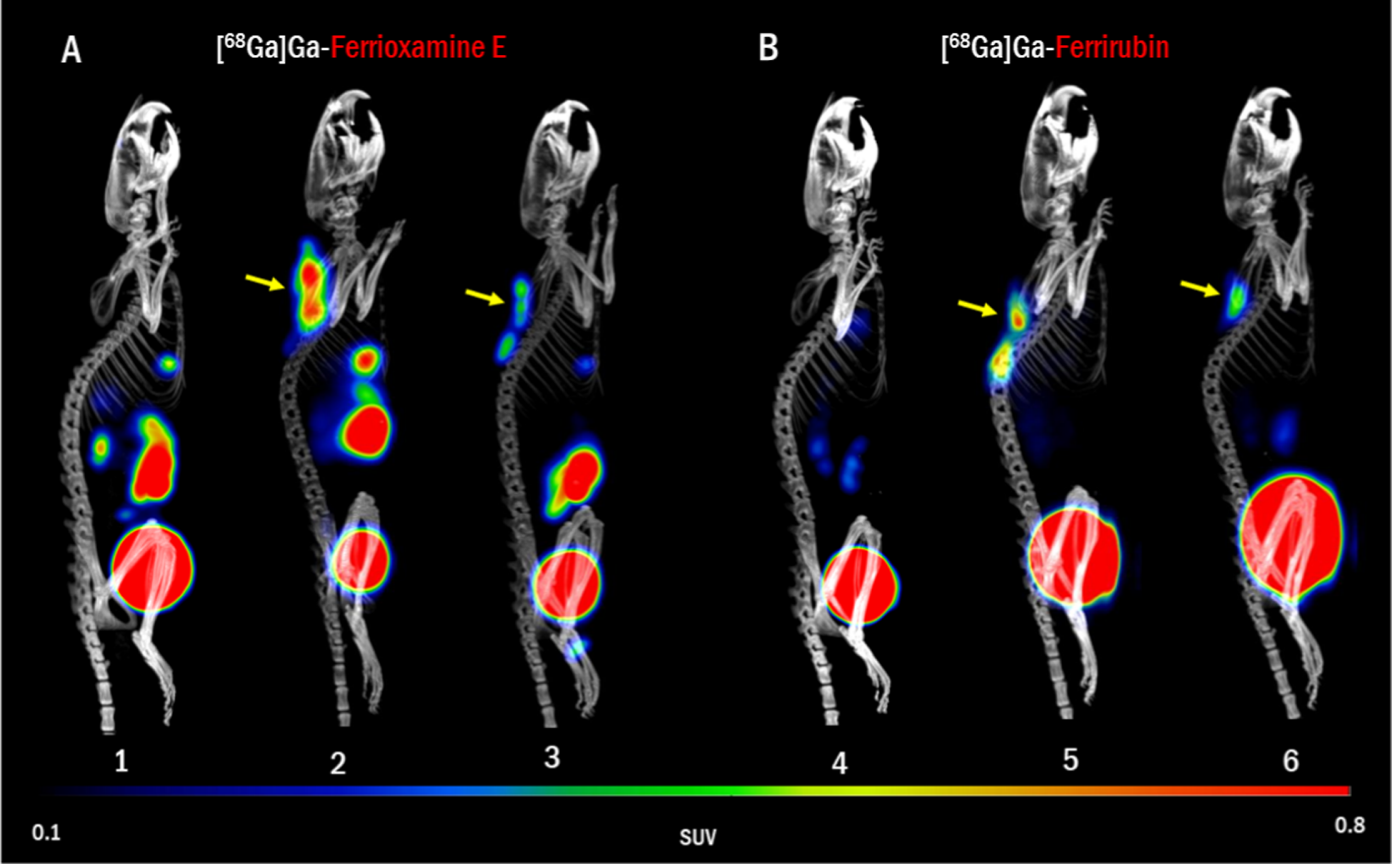
PET/CT in vivo imaging of (A) [^68^Ga]Ga-FOX E and (B) [^68^Ga]Ga-FR in normal mice (1, 4) and in murine model of wound infection induced by *A. baumannii* NCTC 13301 (2–3 and 5–6, yellow arrows). The imaging was performed 24 h after infection and 45 min after radiolabeled siderophore administration. MIP images.

In a murine model of AB-induced dorsal wound infection, we detected signal accumulation with both ^68^Ga-siderophores tested at the site of infection and no accumulation of the signal in the noninfected control animals (Figure 5). Quantitative analysis revealed a significant difference between healthy and infected mice (*P* < 0.001 for [^68^Ga]Ga-FOX E and *P* < 0.05 for [^68^Ga]Ga-FR; Figure S4B).

**Figure 6:**
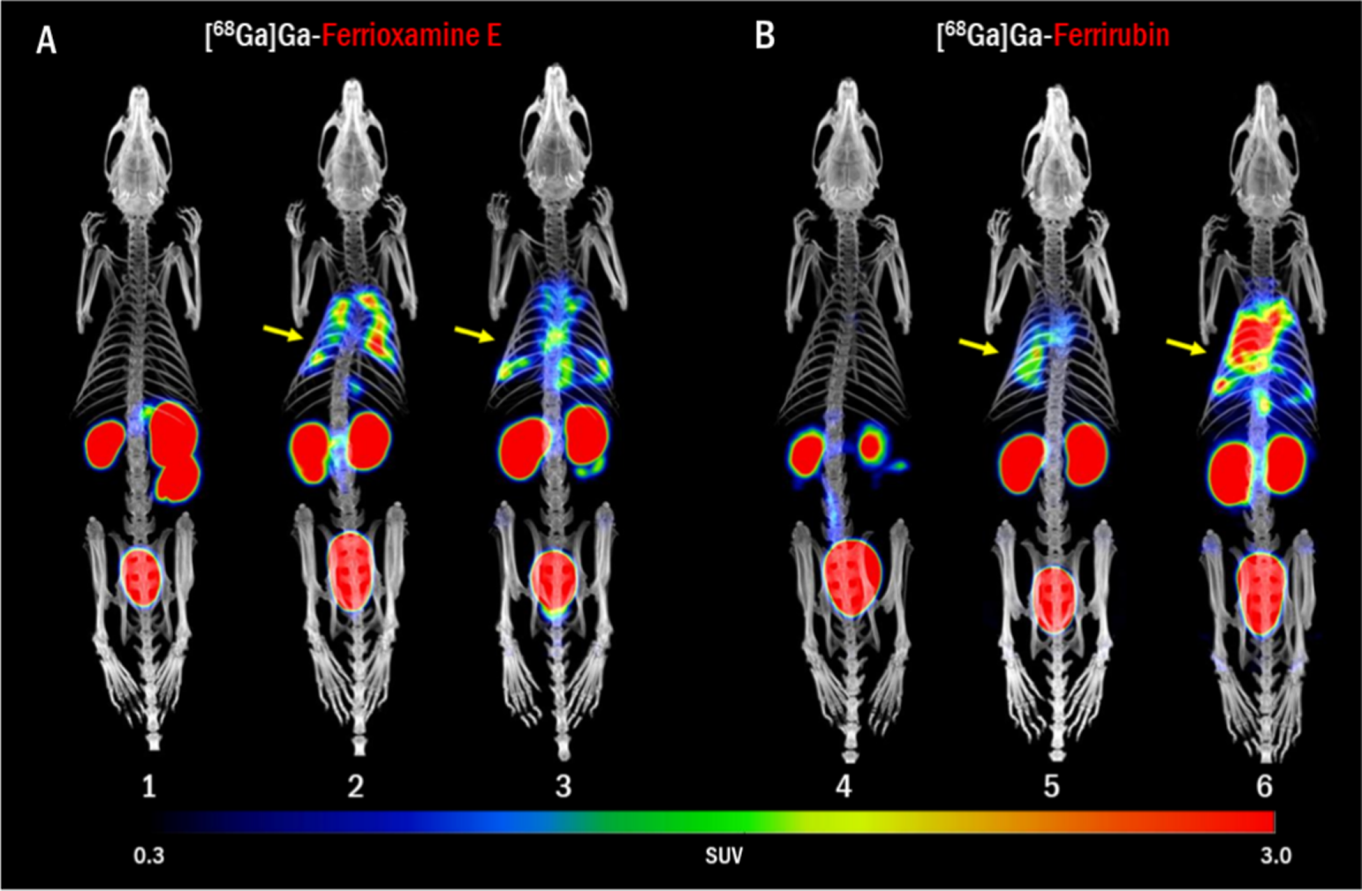
PET/CT in vivo imaging of (A) [^68^Ga]Ga-FOX E and (B) [^68^Ga]Ga-FR biodistribution in control rat (1, 4) and in rat model of lung infection with *A. baumannii* NCTC 13301 (2–3 and 5–6) 48–52 h after infection and 45 min after the injection of radiolabeled siderophore. Yellow arrows indicate the site of infection. MIP images.

**Figure 7:**
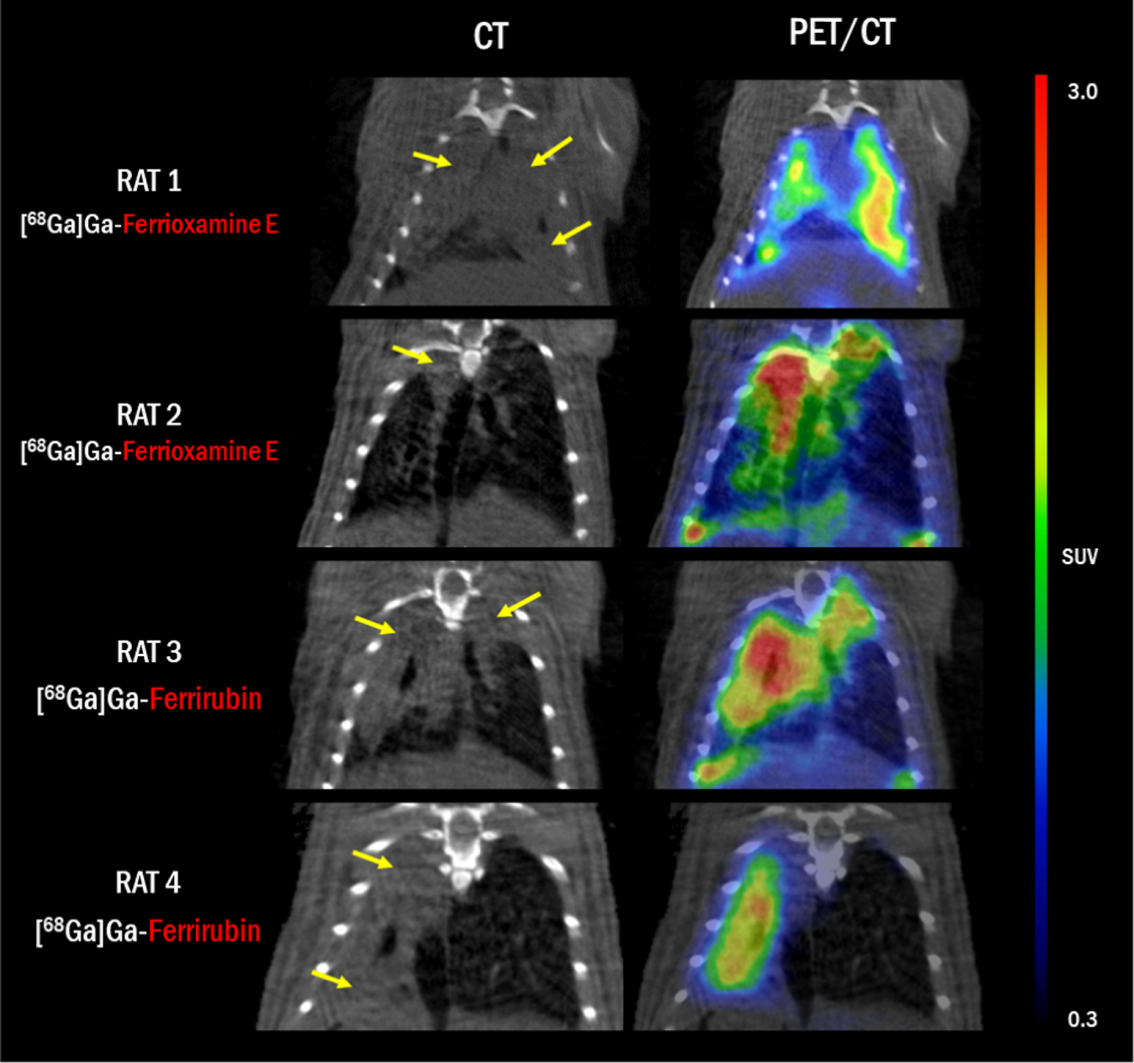
Detailed CT and PET/CT images of coronal sections of lungs of rats with AB-induced pneumonia 48 h after infection and 45 min after the injection of radiolabeled siderophores. Yellow arrows indicate the lesions in the lung tissue.

In the rat model of lung infection, we observed comparably high uptake in the infected lung tissue. The total uptake for [^68^Ga]Ga-FOX E in infected rats was higher than for [^68^Ga]Ga-FR, which also showed a higher background uptake in healthy rats (Figure 6 and 7). Quantitative analysis of the rat pneumonia model revealed a significant difference in mean SUVs between the infected and noninfected rats for [^68^Ga]Ga-FOX E only (*P* < 0.05; Figure S4C).

## Discussion

Despite the advances in medical research, accurate diagnosis of AB infection remains challenging in some cases. Traditional culture-based methods, which are commonly used for diagnosis, can take up to several days to yield results and may be susceptible to bias due to the previous antibiotic use, contamination or the selectivity of the media used.^28^ Although modern culture-independent approaches, such as polymerase chain reaction or next generation sequencing, can rapidly identify a wide variety of pathogens, these methods are highly dependent on correct sampling, have a high risk of sample contamination and may fail to localize the pathogen causing the infection (e.g., upper versus lower respiratory tract infections).^29^ For these reasons, there is an ongoing search for alternative means of detecting bacterial infections. In addition to laboratory methods, imaging techniques are often employed to detect infection. Commonly used methods include X-ray, ultrasound, computed tomography and magnetic resonance imaging. However, these techniques have low sensitivity for detecting infection in its early stages, as structural changes in the tissues are often absent, which can lead to a life-threatening delay in diagnosis in critically ill patients.^30^ Given the fact that functional changes precede the structural changes, there is a great potential for nuclear imaging techniques, such as PET or single photon emission computerized tomography (SPECT), for imaging of infections.^31^ Yet contemporary radiotracers do not meet all the necessary requirements for successful diagnosis of infection. Standard radiopharmaceuticals such as [^18^F]F-fluorodeoxyglucose, [^67^Ga]Ga-citrate or radiolabeled white blood cells are not specific for infection and some are dependent on the host immunity response and might not be optimally used in immunocompromised patients.^32^

Current trends in the development of bacterial imaging tend to focus on tracers that can specifically distinguish between an ongoing bacterial infection and other pathological conditions, which is driving contemporary research in several directions. For example, some radiotracers exploit the host’s immune system (antimicrobial peptides, bacterial-specific antibodies), some take advantage of compounds that already specifically target bacteria (antibiotics, bacteriophages), while others make use of various aspects of bacterial metabolism (nucleoside analogs, d-amino acids, carbohydrates, sugar alcohols, biotin, siderophores).^33,34^

Radiolabeled siderophores have shown promising results in several preclinical studies involving various microorganisms: [^68^Ga]Ga-pyoverdines for imaging of *Pseudomonas aeruginosa* infection, [^68^Ga]Ga-triacetylfusarinine C for imaging of *Aspergillus fumigatus* infection, [^68^Ga]Ga-desferrioxamine B for imaging of various bacterial infections and [^68^Ga]Ga-ornibactin for specific imaging of *Burkholderia cepacia* complex infections.^23−25,35^ Moreover, two clinical trials involving [^68^Ga]Ga-desferrioxamine B for PET imaging in patients with bacterial infections are currently being conducted (EudraCT number: 2020-002868-31; NCT05285072). Here, we explore the possibility of using radiolabeled siderophores for PET imaging of AB infection.

AB produces three structural types of siderophores: acinetobactins and fimsbactins, both of which belong to the mixed catechol-hydroxamate group of siderophores, and baumannoferrins, which are classified as hydroxamates.^36^ Several outer membrane receptors for siderophore uptake have been identified in AB. However, the most important receptor involved in the uptake of xenosiderophores appears to be the FhuE receptor.^21,37,38^ This outer membrane receptor is energetically dependent on the TonB system.^39^ Siderophores are transported into the periplasmic space, from where they are imported into the cytoplasm by the ABC complex driven by ATP hydrolysis. Once in the cytoplasm, iron is released from the siderophore by reduction of iron.^40^ Proteomic analysis published by Tiwari et al. demonstrated, that the FhuE receptor might be capable of binding 31 out of the 33 tested xenosiderophores. However, the study also suggests, that the FhuE receptor is not capable of docking FOX E, which is contradiction to our results, suggesting the potential involvement of an alternative receptor in the uptake of FOX E.^38^

We successfully radiolabeled all siderophores with high radiochemical purity, with the only exception of [^68^Ga]Ga-FR, which reached values lower than the other siderophores. On the radio RP-HPLC we observed the main peak of [^68^Ga]Ga-FR, that exceeded 91%. We also observed a presence of two small peaks, shortly preceding the main peak. According to Krasulova et al., this might indicate a presence of other FR isomers or different types of ferrichrome siderophores.^27^ The first in vitro experiment in this study, evaluating the uptake of various radiolabeled siderophores, showed that AB is able to utilize several siderophores and that radiolabeling with gallium-68 does not interfere with their uptake into the bacterial cell. A few siderophores, however, showed very low uptake in AB in both cultivation media. Specifically, ORNB, siderophore produced by *B. cepacia* complex, requires highly specific outer membrane receptor *orbA*, which is not present in AB.^38,41,42^ Similarly, siderophore SAL S4, utilized by *Enterobacteriaceae*, is taken up by the IroN receptor, which AB lacks.^42,43^ Additionally, the fungal siderophore FCHA, which does not function as an ionophore in vivo but is hypothesized to act solely as an iron carrier to the outer bacterial membrane, also showed no uptake in AB.^44^ Despite the expectation that these siderophores would not demonstrate any uptake in AB, they were included in the study to serve as a form of negative control.

For the siderophores that demonstrated some uptake levels in AB, several factors were considered in selecting the most appropriate siderophores for further testing: The selected siderophore should (1) be easy to radiolabel with high radiochemical purity, (2) have high uptake in M9 medium, which simulates the environment during the infectious process and the iron-free conditions stimulate the bacteria to express siderophore receptors^16,45^ (3) have at least moderate level of uptake in MH medium to demonstrate its in vitro uptake in standard medium and that it is not completely dependent on the environment^46^ (4) have good pharmacokinetic properties. Overall, we observed low levels of uptake in cultures grown in MH medium for several ^68^Ga-siderophores and very high uptake for the majority of ^68^Ga-siderophores tested in cultures grown in M9 medium. We decided to exclude ferrichrome, ferricrocin and ferrichrysin from further testing, as they do not have favorable pharmacokinetic properties as has been previously described.^22^ Although enterobactin, coprogen, desferrioxamine B and aerobactin all showed decent uptake in M9 medium, their negligible uptake in MH medium led us to withdraw them from subsequent experiments.

Based on the obtained results, we selected two hydroxamate siderophores, ferrioxamine E and ferrirubin, which both met our specified requirements. Both ^68^Ga-siderophores have favorable in vitro and in vivo properties, such as low plasma protein binding values, hydrophilicity and stability in human serum, as evaluated in previous studies.^26,27^ Although both siderophores are classified as hydroxamates, like some AB-produced siderophores, there is a difference in the species that produce them. Ferrioxamine E is a bacterial siderophore produced by *Streptomyces olivaceus* and ferrirubin is a fungal siderophore isolated from *Aspergillus ochraceus*.^47^

We have shown that both ^68^Ga-siderophores have high and comparable uptake in different AB strains from clinical samples. These results indicate that both ^68^Ga-siderophores have the potential to be used for the diagnosis of wide range of AB infections. We also demonstrated that ^68^Ga-siderophore uptake in AB is an active process requiring live bacteria, as no uptake was observed in heat-inactivated cultures. In AB cultures preincubated with excess of iron, we observed a decrease in uptake for both ^68^Ga-siderophores, but it was not completely blocked. The situation was different in cultures preincubated with Fe-siderophore complex. The uptake of both ^68^Ga-siderophores was completely blocked in AB culture that was preincubated with Fe-FOX E. On the other hand, in the culture, that was preincubated with Fe-FR, only the uptake of [^68^Ga]Ga-FR was completely blocked, but [^68^Ga]Ga-FOX E retained a small level of uptake into the bacterium. This might be indicative of either nonspecific binding of [^68^Ga]Ga-FOX E or the interaction with an alternative receptor that might bind FOX-E but not FR. This phenomenon has been observed in other pathogens previously.^22,48^ Surprisingly, we found that the uptake of ^68^Ga-siderophores did not increase in time and reached high levels at the first time point observed, contrary to what we have observed with other ^68^Ga-siderophores in previous experiments.^22,35^ However, it has to be taken into account, that the tested AB culture was grown in minimal media, where the iron-limited conditions strongly upregulate the genes coding transport of siderophores.^49^

In vivo PET/CT imaging in normal mice showed that both ^68^Ga-siderophores have rapid pharmacokinetics and neither showed excessive accumulation in major organs. However, [^68^Ga]Ga-FR exhibits superior biodistribution due to its exclusive urinary excretion. In contrast, [^68^Ga]Ga-FOX E is eliminated from the mouse body through both the urinary and gastrointestinal systems, resulting in significant activity in the gallbladder and intestine, which may complicate the localization of gastrointestinal infections using PET/CT imaging. PET/CT imaging studies in all animal models of infection showed that both ^68^Ga-siderophores had comparably high uptake in infected tissue and neither ^68^Ga-siderophore showed uptake in any of the noninfected control animals. This demonstrates, that both ^68^Ga-siderophores can be used to image different sites of infection. In a dynamic study, both radiotracers were able to rapidly localize the site of infection as early as 5 min after the infection, an important characteristic for a radiotracer according to Ordonez et al.^33^ When different doses of bacteria were imaged, only [^68^Ga]Ga-FOX E was barely able to reach the detection level of 10^4^ CFU, that is the required threshold for the diagnosis of ventilator-associated pneumonia using bronchoalveolar lavage.^50,51^ However, in cases of infection, sputum and tracheal aspirates usually yield more than 10^5^ CFU/ml, a number of bacteria that both ^68^Ga-siderophores were able to detect.^52^ The quantification study showed, that [^68^Ga]Ga-FOX E generally had a greater statistical difference between infected and noninfected animals than [^68^Ga]Ga-FR in all models and that [^68^Ga]Ga-FR had a higher background signal in control animals.

## Conclusion

In this work, we demonstrated that radiolabeling does not interfere with siderophore uptake into the bacterial cell and that AB can utilize a variety of ^68^Ga-siderophores. We selected the two most promising siderophores radiolabeled with Ga-68 and evaluated their in vitro uptake into AB cultures and in vivo biodistribution. The results suggest that both ^68^Ga-siderophores can be used to diagnose AB infection, as both ^68^Ga-siderophores have favorable in vitro properties and proved their versatility by displaying high accumulation in infected tissues in three animal models of infection induced by AB: murine model of myositis, murine model of dorsal wound infection and rat model of pneumonia. Although [^68^Ga]Ga-FR has a better biodistribution in terms of organ uptake in healthy animals, it also has a higher background signal. [^68^Ga]Ga-FOX E showed superior results in quantification studies, exhibiting a statistically significant difference between control and infected animals in all animal models. These results suggest that radiolabeled siderophores may have possible applications in the diagnosis, localization and therapy monitoring of AB infections.

## Methods

### Chemicals, Reagents and Siderophores

Chemicals and reagents for the study were purchased as reagent grade from commercial sources without further purification. All siderophores used in the study were purchased from Biophore Research Products (Tübingen, Germany), except for Desferal, which was purchased from Novartis (Basel, Switzerland). The ^68^GaCl_3_ used for radiolabeling was obtained from a ^68^Ge/^68^Ga-generator (Eckert & Ziegler Eurotope GmbH, Berlin, Germany) using a fractionated elution method with 0.1 M HCl.^53^

## Radiolabeling of FR and FOX E

The reaction mixture for FR was prepared by mixing 5 μg of FR dissolved in water (1 μg/μL) with 30 μL of sodium acetate (155 mg/mL in water) and 300 μL of ^68^GaCl_3_ generator eluate (25–40 MBq). This mixture was incubated at RT for 5 min.

The reaction mixture for FOX E was prepared by mixing 20 μg of FOX E dissolved in 10% ethanol (1 μg/μL) with 30 μL of sodium acetate (155 mg/mL in water) and 300 μL of ^68^GaCl_3_ generator eluate (25–40 MBq). This mixture was incubated at 80 °C for 20 min.

After incubation, the pH of both siderophores was adjusted to 5–6 by the addition of 100 μL of sodium acetate. The radiochemical purity of the final products (Figure 1) was analyzed by either radio reversed-phase high-performance liquid chromatography (radio-RP-HPLC) or radio instant thin-layer chromatography (radio-iTLC), as described below.

### Quality Control of Radiolabeled Siderophores

The radiochemical purity of the radiolabeled siderophores was evaluated using the radio-RP-HPLC gradient method^35^ (Dionex UltiMate 3000, Thermo Scientific, Waltham, MA, USA) in combination with a radiometric detector (GABI Star, Raytest, Straubenhardt, Germany). A column (Nucleosil 120-5 C18 250 × 40 mm, WATREX, Prague, Czech Republic) with a flow rate of 1 mL/min, oven temperature of 25 °C and ultraviolet detection at 225 and 250 nm was used with acetonitrile (ACN)/0.1% trifluoroacetic acid (TFA)/H_2_O as the mobile phase with the following gradient: 0–2 min −0% ACN; 2–15 min −0–36% ACN; 15–18 min −36–60% ACN; 18–19.5 min −60% ACN; 19.5–20 min −60–0% ACN; 20–24 min −0% ACN.

Additional evaluation of the radiochemical purity of radiolabeled siderophores was performed by radio-iTLC using silica gel impregnated glass microfibre chromatographic papers (Varian, Lake Forest, CA, USA). ^68^Ga-siderophore complex samples were applied to the chromatographic paper strips which were then developed in a chamber saturated with equal parts of ammonium acetate (1 M) and methanol. After development of the samples, the strips were scanned using a radiometric Phosphor Imager (Cyclone Plus Storage Phosphor System, PerkinElmer, Waltham, MA, USA), and the chromatograms for each strip were evaluated and quantified using the OptiQuant software (PerkinElmer, Waltham, MA, USA).

### Microbial Strains and Growth Conditions

The list of microbial strains used in this study can be found in Table ST1. The bacterial strains were first cultured on Petri dishes containing solid medium of Columbia blood agar medium for 24 h at 30 °C. The bacteria were then transferred to Erlenmeyer flasks containing either 10 mL of M9 minimal salts medium with 1% casamino acids (M9) or 10 mL of Mueller–Hinton broth (MH). The flasks were shaken at 120 rpm for 16–24 h. The quantification of bacteria was performed by measuring the absorbance at 600 nm using a table photometer (DEN-600 Photometer, Biosan, Latvia) and calculating the amount of colony forming units from the standard curve for each bacterial strain.

### In Vitro Uptake Assays

For the in vitro uptake assays, siderophores (*c* ∼ 200 nM) were incubated with AB strains under various conditions in Eppendorf tubes that were shaken at 300 rpm for 45 min at 37 °C. After the incubation, the uptake was interrupted by centrifugation at 15 000 rpm for 5 min, removal of the supernatant and rinsing of the microbial sediment with ice-cold Tris buffer (10 mM tris(hydroxymethyl)aminomethane in 0.9% NaCl). After rinsing, the tubes containing the microbial sediment were weighed and measured on a γ-counter (2480 Wizard^2^ automatic gamma counter; PerkinElmer, Waltham, MA, USA). The results were expressed as the percentage of applied dose per gram of microbial culture (% AD/g).

To further evaluate the uptake of radiolabeled siderophores by AB, several assays were performed. (i) To determine which siderophores can be used by AB, various radiolabeled siderophores were incubated with AB NCTC 13301 grown in M9 or MH and handled as described above. (ii) To investigate the uptake of [^68^Ga]Ga-FOX E and [^68^Ga]Ga-FR in different AB strains, both siderophores were incubated with various clinical samples of AB and handled as described above. (iii) To demonstrate specific and active uptake of ^68^Ga-siderophores, the first AB NCTC 13301 culture was heated at 90 °C for 20 min, the second culture was preincubated with 50 μL of 0.1 M FeCl_3_ (37 °C, 300 rpm, 20 min), the third culture was preincubated with Fe-FOX E (37 °C, 300 rpm, 20 min) and the fourth culture was preincubated with Fe-FR (37 °C, 300 rpm, 20 min), after which all the cultures were incubated with [^68^Ga]Ga-FOX E or [^68^Ga]Ga-FR and handled as above. (iv) To estimate the uptake of radiolabeled siderophores over time, the normal AB NCTC 13301 and AB NCTC 13301 culture preincubated for 20 min with 50 μL 0.1 FeCl_3_ used as a control culture were incubated with [^68^Ga]Ga-FOX E or [^68^Ga]Ga-FR for 5, 15, 30, 45, 60, and 90 min, after which the samples were handled as described above.

### Animal Experiments

Female 8–10 week old Balb/c mice and female 8–10 week old Lewis rats (Envigo, Horst, The Netherlands) were used for animal experiments in this study. All animals were acclimatized to laboratory conditions at least for 1 week prior to the experiments. Animals were housed under standard laboratory conditions on sawdust, in individually ventilated cages and with free access to animal chew and water. General health and body were monitored throughout the experiments. The number of experimental animals used for all in vivo experiments was reduced as much as possible (usually *n* = 3–4 per group and time point). To avoid animal suffering and to reduce movement artifacts, injections, administrations of bacterial infection and imaging studies were performed under 2% isoflurane anesthesia (FORANE, Abott Laboratories, Abbott Park, IL, USA). All animal experiments were conducted in accordance with regulations and guidelines of the Czech Animal Protection Act (no. 246/1992), and with the approval of the Czech Ministry of Education, Youth, and Sports (MSMT-24421/2021-4) and the institutional Animal Welfare Committee of the Faculty of Medicine and Dentistry of Palacký University in Olomouc.

### Animal Infection Models

The murine model of acute myositis was performed in mice immunosuppressed by intraperitoneal (i.p.) injection of cyclophosphamide (Endoxan, Baxter, Prague, Czech Republic) five and 1 day before infection (receiving 150 and 100 mg/kg doses, respectively). On the day of infection, all mice were intramuscularly (i.m.) injected with 50 μL of bacterial culture containing AB NCTC 13301 (*V* = 50 μL, dose ∼10^4^–10^6^ CFU) into the muscle of the left hind leg. To test the in vivo specificity of radiolabeled siderophores for active infection, 50 μL of AB NCTC 13301 bacterial culture (live or heat-inactivated at 90 °C for 20 min), saline or turpentine oil (24 h prior to imaging, to induce sterile inflammation) was injected into the right hind leg muscle. Microbial infections were allowed to develop for 5 h for imaging studies.

The murine dorsal wound infection model was performed according to Thompson et al. in mice immunosuppressed as described above.^54^ Birefly, on the day of infection, mice were placed in the prone position under isoflurane anesthesia. The mice were hair clipped and scrubbed with iodine solution from cervical to lumbar dorsum. A full-thickness skin incision was made in the area over the thoracic spinal using a 6.0 mm disposable skin biopsy punch (Disposable biopsy punch, Kai industries co., Itd., Seki, Japan). Bacterial culture of AB NCTC 13301 (*V* = 25 μL, dose = 5 × 10^4^ CFU) was pipetted directly into the wound and allowed to absorb for 2 min. The wound was covered with a sterile dressing (Tegaderm, Deutschland GmbH, Neuss, Germany) and secured with tissue adhesive (Surgibond, SMI AG, Steinerberg, Belgium). At the end of the procedure, the mice received a dose of 0.05 mg/kg buprenorphine (i.m.) for pain management. Microbial infection was allowed to develop for 24 h for static imaging studies.

The rat model of pneumonia was performed in rats immunosuppressed by i.p. injection of cyclophosphamide five and 1 day prior to the infection (75 mg/kg). Rats under isoflurane anesthesia were infected by intratracheal administration of 100 μL of AB NCTC 13301 culture (V = 100 μL, dose = 10^6^ CFU) using Tele Pack Vet x Led system equipped with a rigid endoscope (Karl Storz GmbH & Co. KG, Tuttlingen, Germany). Microbial infection was allowed to develop for 48–52 h for static imaging studies.

### Animal Imaging Studies

Experimental animals were placed under inhalation anesthesia using isoflurane and were retro-orbitally (r.o.) injected with either ∼5 μg of [^68^Ga]Ga-FOX E or ∼1 μg of [^68^Ga]Ga-FR at a dose of 5–10 MBq per animal. Animals were placed in the prone position in the Mediso NanoScan PET/CT small animal imaging system (Mediso Medical Imaging Systems, Budapest, Hungary). Static imaging was performed 30 and 90 min after administration of radiolabeled siderophore for imaging studies in healthy animals or 45 min after administration of radiolabeled siderophore for imaging studies in infected animals. A dynamic imaging study was initiated <5 min p.i. Single FOV (98,5 mm) for mice and double FOV (2 × 98.5 mm) for rats PET scans were performed, immediately followed by a whole body helical CT scan (50 kVp/980 μA, 720 projections). Image reconstruction was performed using Mediso Tera-Tomo 3D PET iterative reconstruction (Mediso Medical Imaging Systems, Budapest, Hungary). Image visualization, analysis, processing and quantification were performed in Mediso InterView FUSION (Mediso Medical Imaging Systems, Budapest, Hungary). All scans were normalized to injected activity and animal weight.

Quantitative analysis of the myositis (both static and dynamic), dorsal wound and pneumonia models was performed by measuring standardized uptake value (SUV) within a region of interest (ROI). The ROIs were drawn based on the anatomical structures visualized by CT scans. For myositis model, both infected and noninfected mouse hind legs were measured and compared separately. In addition to the dynamic study, time–activity curves were generated for both radiolabeled siderophores, comparing the amount of activity in healthy and infected legs over time. For the dorsal wound model, the soft tissues of the dorsal region above the thoracic spine of normal healthy mice as controls and mice with AB-induced dorsal wound infection were measured. For the pneumonia model, the whole lung region excluding the heart and major arteries was measured in normal control rats and in rats with AB-induced pneumonia. The results were expressed as SUV_mean_.

## Data Availability

Statistical analyses were performed in Microsoft Office 365 Excel (Microsoft Corporation, Redmond, WA, USA). Data were analyzed using an unpaired two-tailed Student’s *t*-test. All graphs presented include error bars representing the standard deviation. Other data, including the in vitro uptake of radiolabeled siderophores, are reported as the mean value ±standard deviation.

## Supporting Information Available

Quality control of [^68^Ga]Ga-FOX E and [^68^Ga]Ga-FR; PET/CT in vivo imaging in AB induced myositis model with various infectious doses; PET in vivo dynamic study of AB induced myositis model and time–activity curves; quantification of radioactive signal uptake in three models of AB-induced infection; list of microbial strains used in the study (PDF)

## Abbreviations USED

^68^Ga: gallium-68
AB: *Acinetobacter baumannii*
ACN: acetonitrile
AERO: [^68^Ga]Ga-aerobactin
COP: [^68^Ga]Ga-coprogen
CT: computed tomography
DFO: [^68^Ga]Ga-desferrioxamine B
ENTB: [^68^Ga]Ga-enterobactin
FC: [^68^Ga]Ga-ferricrocin
FCH: [^68^Ga]Ga-ferrichrome
FCH A: [^68^Ga]Ga-ferrichrome A
FCHR: [^68^Ga]Ga-ferrichrysin
FOX E: ferrioxamine E
FR: ferrirubin
HAI: hospital acquired infections
i.m.: intramuscular
i.p.: intraperitoneal
M9: M9 minimal salts medium
MH: Mueller–Hinton broth
MIP: maximum intensity projection
ORNB: [^68^Ga]Ga-ornibactin
PET: positron emission tomography
p.i.: post injection
radio-iTLC: radio instant thin-layer chromatography
radio-RP-HPLC: radio reversed-phase high-performance liquid chromatography
r.o.: retro-orbitally
ROI: region of interest
RT: room temperature
SAL S4: [^68^Ga]Ga-salmochelin S4
SPECT: single photon emission computerized tomography
SUV: standardized uptake value
TFA: trifluoroacetic acid
