# Supplementary material for: Positron Emission Tomography Imaging of Acinetobacter baumannii Infection: Comparison of Gallium-68 Labeled Siderophores
Source: ACS Infect Dis. 2025 Mar 18;11(4):917–28. doi: 10.1021/acsinfecdis.4c00946 (PMC11997986; doi:10.1021/acsinfecdis.4c00946)
Supplement: Supplementary file 1 — id4c00946_si_001.pdf [file id4c00946_si_001.pdf]

SUPPORTING INFORMATION

POSITRON EMISSION TOMOGRAPHY IMAGING OF ACINETOBACTER BAUMANNII INFECTION: COMPARISON OF GALLIUM-68 LABELED SIDEROPHORES

Katerina Dvorakova Bendova[1], Kristyna Krasulova[1], Barbora Neuzilova[1], Miroslav Popper[1], Patrik Mlynarcik[2], Katarina Hajduova[1], Zbynek Novy[1,4], Marian Hajduch[1,3,4], and Milos Petrik[1,3,4*]

1 Institute of Molecular and Translational Medicine, Faculty of Medicine and Dentistry, Palacký University, 779 00, Olomouc, Czech Republic

2 Department of Microbiology, Faculty of Medicine and Dentistry, Palacký University and University Hospital, 775 15, Olomouc, Czech Republic

3 Laboratory of Experimental Medicine, University Hospital, 779 00, Olomouc, Czech Republic

4 Czech Advanced Technology and Research Institute, Palacký University, 779 00, Olomouc, Czech Republic

*Corresponding author: Milos Petrik - Institute of Molecular and Translational Medicine, Hnevotinska 5, CZ-77900 Olomouc, Czech Republic, Tel: +420585632126; Fax: +420585632180; Email: milos.petrik@upol.cz, https://orcid.org/0000-0003-1334-5916

TABLE OF CONTENTS

Figure S1. Quality control of [^{68}Ga]Ga-FOX E and [^{68}Ga]Ga-FR S2

Figure S2. PET/CT *in vivo* imaging in AB induced myositis model with various infectious doses S4

Figure S3. PET *in vivo* dynamic study AB induced myositis model and time-activity curves S5

Figure S4. Quantification of radioactive signal uptake in three models of AB-induced infection. S7

Table S1. List of microbial strains used in the study S8

Figure S1: Quality control of [^{68}Ga]Ga-FOX E and [^{68}Ga]Ga-FR performed on **(A)** RP-radioHPLC and **(B)** radio-iTLC-SG.

A

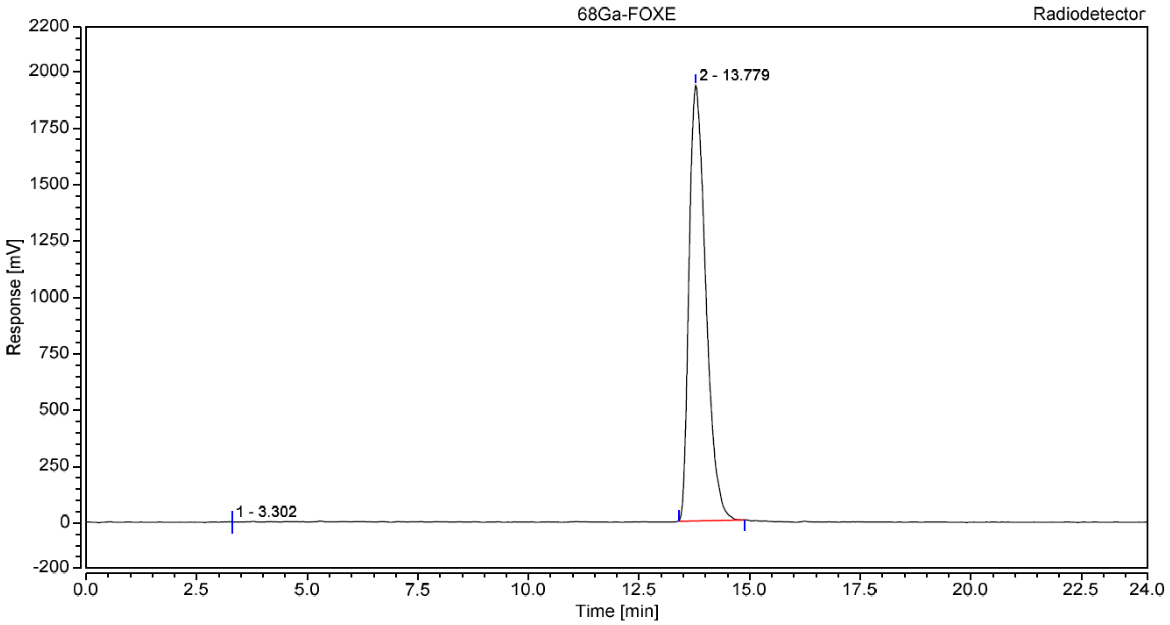

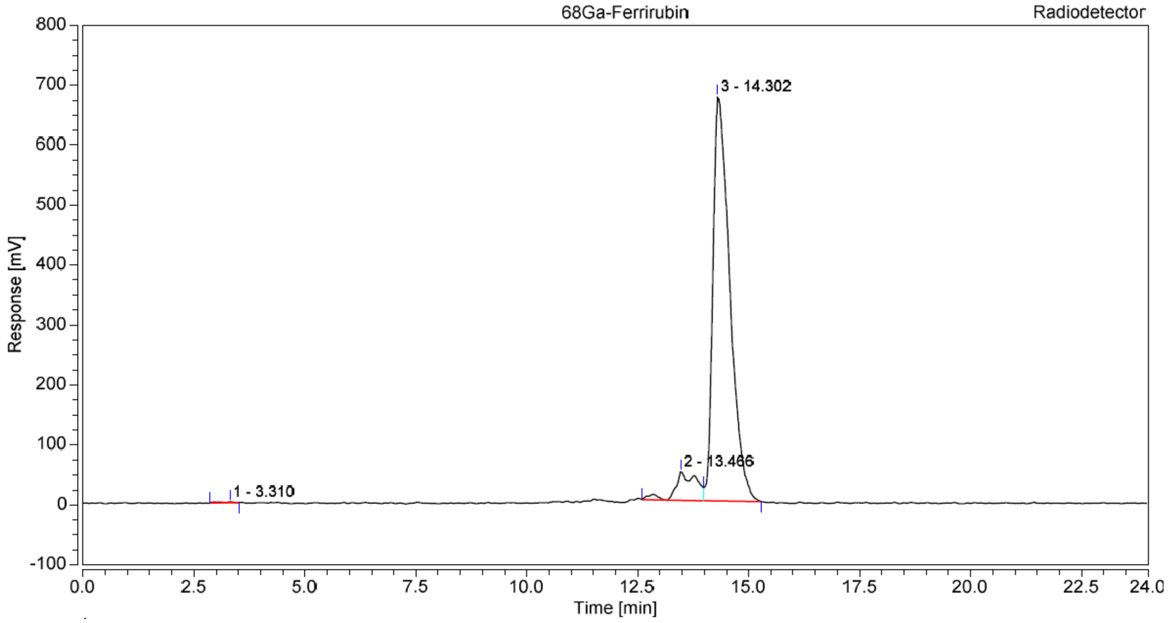

B

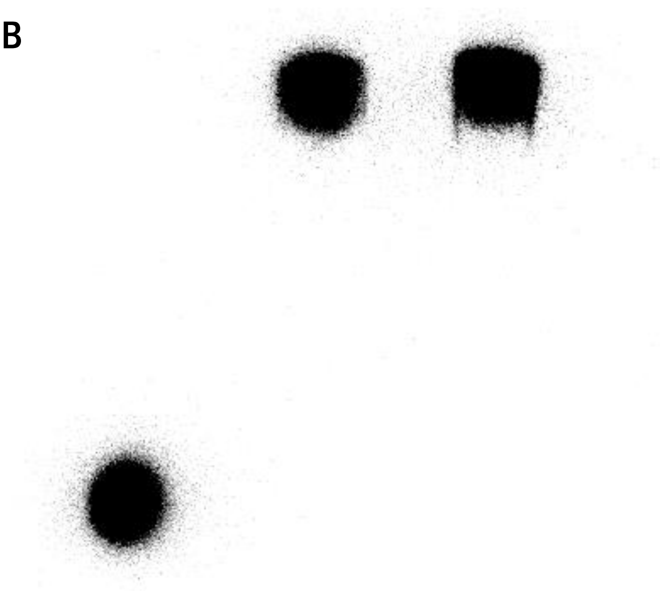

Figure S2: PET/CT *in vivo* imaging of (A) [^{68}Ga]Ga-FOX E and (B) [^{68}Ga]Ga-FR biodistribution in the AB-induced myositis model in mice 5 h after infection and 45 min after administration of radiolabeled siderophore (maximum intensity projection images). Each mouse recieved a different amount of bacteria: (1) dose = 8x10^6 CFU; (2) dose = 8x10^5 CFU; (3) dose = 8x10^4 CFU. The yellow arrow indicates the site of infection.

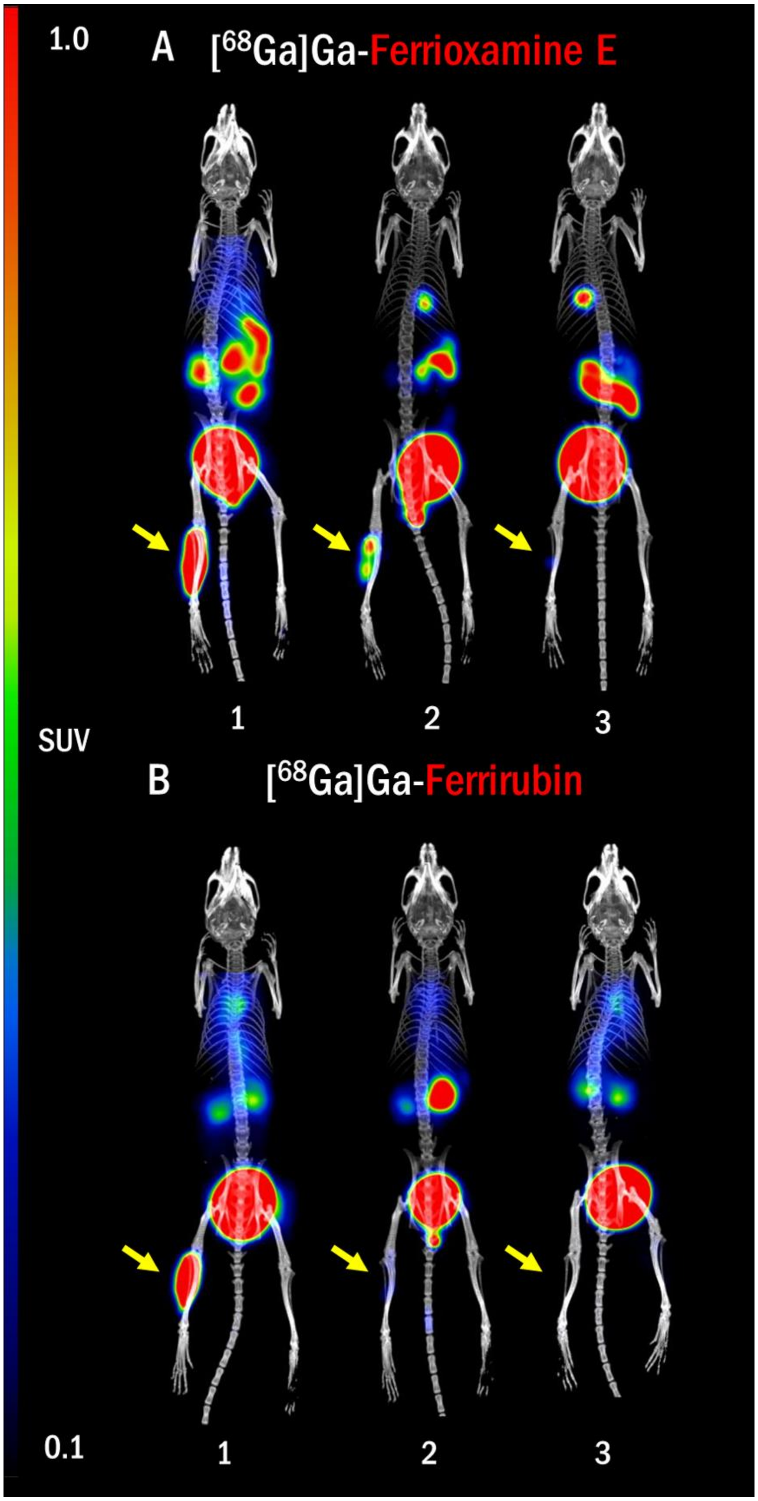

Figure S3: **(A)** PET/CT dynamic *in vivo* biodistribution study of **(A1)** [^{68}Ga]Ga-FOX E and **(A2)** [^{68}Ga]Ga-FR in mice with AB muscle infection in left hind leg 5 hours after infection and 5-60 min after radiolabeled siderophore administration (MIP images). H = heart, K = kidneys, B = bladder, **INF** = site of infection (yellow). **(B)** Time-activity curves for mice in dynamic study shown in Figure S3A, comparing the activity in infected leg (red line) to healthy leg (black line). **(B1)** Time activity curve for [^{68}Ga]Ga-FOX E. **(B2)** Time activity curve for [^{68}Ga]Ga-FR.

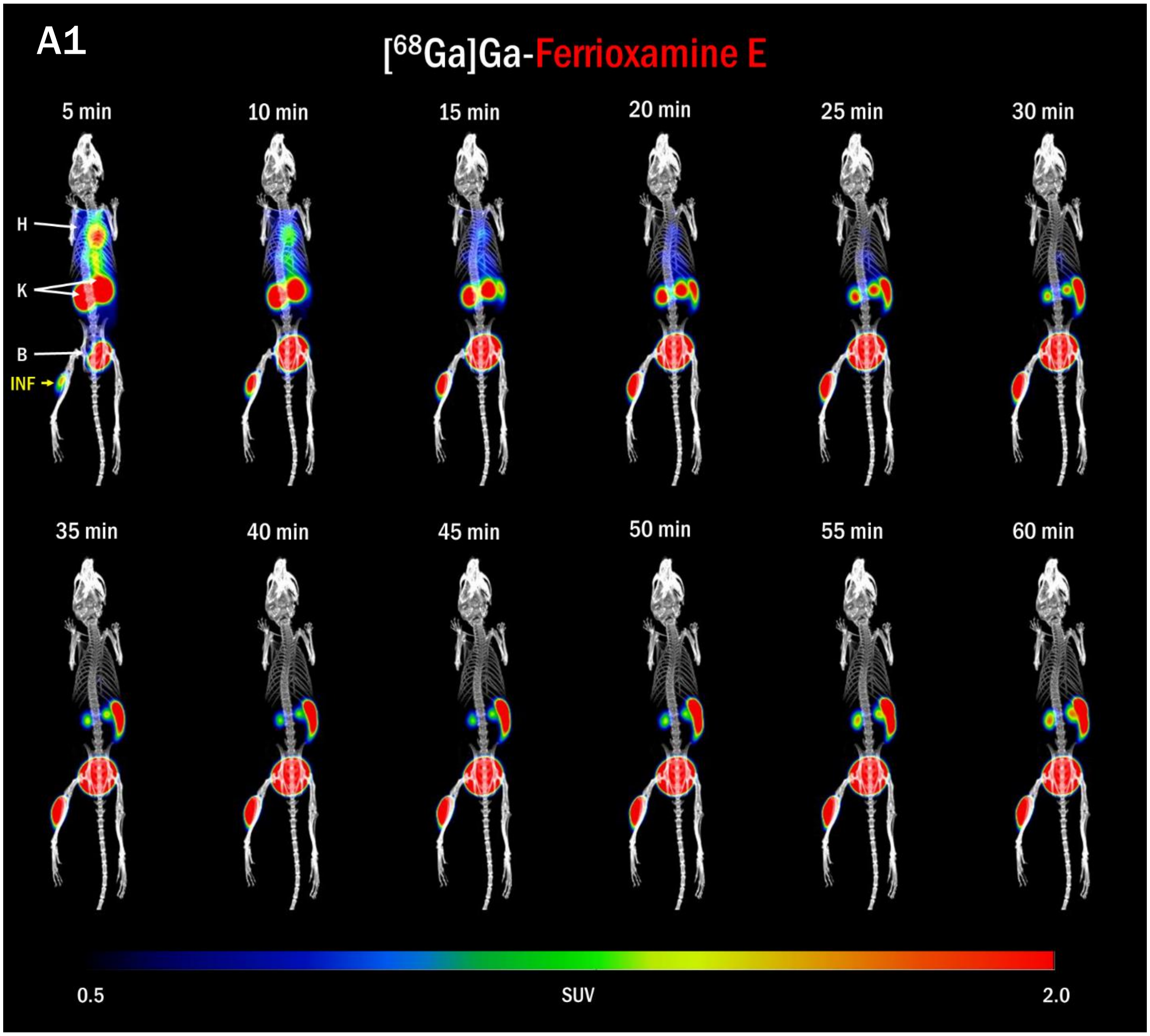

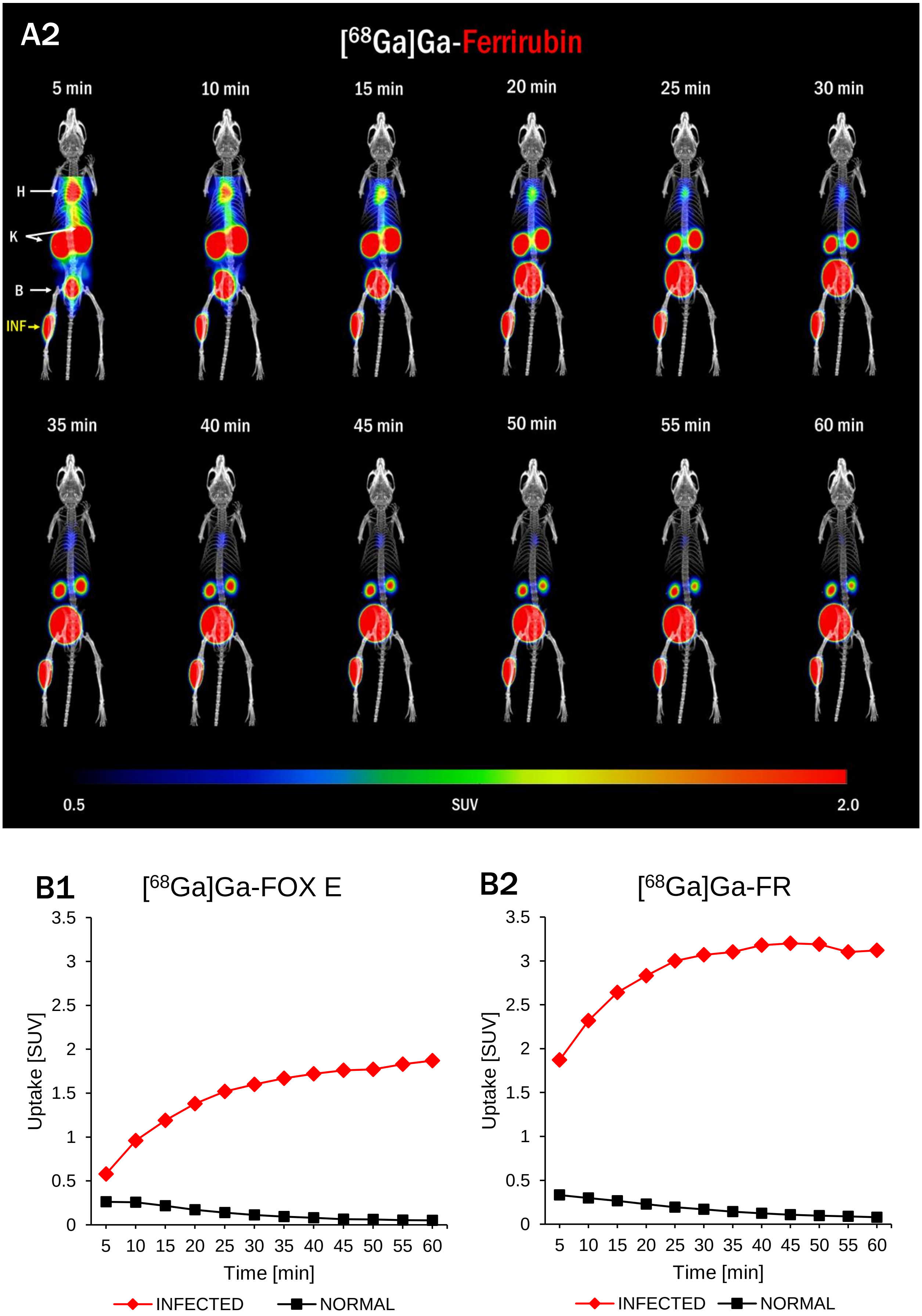
A2
[68Ga]Ga-Ferrirubin
5 min
10 min
15 min
20 min
25 min
30 min
H
K
B
INF
35 min
40 min
45 min
50 min
55 min
60 min
0.5
SUV
2.0
B1
[68Ga]Ga-FOX E
Uptake [SUV]
0 0.5 1 1.5 2 2.5 3 3.5
5 10 15 20 25 30 35 40 45 50 55 60
Time [min]
INFECTED
NORMAL
B2
[68Ga]Ga-FR
Uptake [SUV]
0 0.5 1 1.5 2 2.5 3 3.5
5 10 15 20 25 30 35 40 45 50 55 60
Time [min]
INFECTED
NORMAL

Figure S4: Quantification of radioactive signal uptake in three models of AB-induced infection. **(A)** Comparison of the mean signal uptake in infected to uninfected legs of mice in a model of murine myositis (n = 3). **(B)** Comparison of the mean signal uptake in the dorsal area of healthy mice and the mice with infected wounds. **(C)** Comparison of the mean signal uptake in the lungs of uninfected rats and rats with pneumonia (n=3). Results are expressed as standardized uptake value (SUV); **P* < 0.05; ***P < 0.01;* ****P* < 0.001.

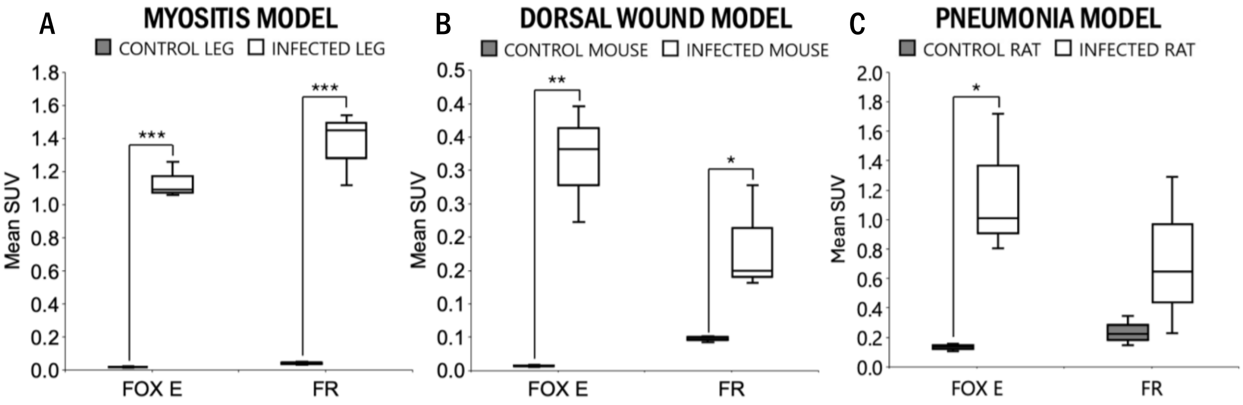

Table S1: List of microbial isolates from clinical samples used in the study. Isolates were obtained from the collection of the Department of Microbiology, Faculty of Medicine and Dentistry, Palacký University Olomouc.

	**Species**	**Isolate number**	**Origin**
AB1	*Acinetobacter baumannii*	9022/c	endotracheal secretion, 21-year-old man, coma vigile, suspected colonization
AB2	*Acinetobacter baumannii*	7948/c	endotracheal secretion, 57-year-old woman, patient with chronic obstructive pulmonary disease
AB3	*Acinetobacter baumannii*	6535/a	swab from the wound on the ankle, a 27-year-old Ukrainian man, injured after a mine explosion
AB4	*Acinetobacter baumannii*	11069/a	swab from a gunshot wound on the head, a 61-year-old man
AB5	*Acinetobacter baumannii*	8905/c	endotracheal secretion, 51-year-old man, hospital-acquired pneumonia, hospitalized for craniotrauma
AB6	*Acinetobacter baumannii*	13515/a	endotracheal secretion, 22-year-old man, coma vigile, pneumonia
AB7	*Acinetobacter baumannii*	17807/a	urine from a permanent urinary catheter, 24-year-old man, coma vigile, urinary tract infection
AB8	*Acinetobacter baumannii*	20192/c	sputum, 86-year-old woman, patient with chronic obstructive pulmonary disease, COVID-19 positive
